# Risk factors and nomogram prediction model for isolated distal deep vein thrombosis after endovascular treatment in acute ischemic stroke patients

**DOI:** 10.3389/fneur.2026.1701326

**Published:** 2026-02-24

**Authors:** Chao Wei, Xiang Li, Yueshi Huang, Renshuai Liu, Yu Yao, Lintao Zhou, Chengjie Jiang, Jinghui Lin, Jianhong Yang

**Affiliations:** 1 Centre of Cerebrovascular Diseases, The First Affiliated Hospital of Ningbo University, Ningbo, Zhejiang, China; 2 Department of Neurology, The First Affiliated Hospital of Ningbo University, Ningbo, Zhejiang, China; 3 Department of Intensive Care Unit, The First Affiliated Hospital of Ningbo University, Ningbo, Zhejiang, China; 4 Department of Neurosurgery, The First Affiliated Hospital of Ningbo University, Ningbo, Zhejiang, China; 5 Ningbo Key Laboratory of Nervous System and Brain Function, The First Affiliated Hospital of Ningbo University, Ningbo, Zhejiang, China

**Keywords:** acute ischemic stroke, endovascular treatment, isolated distal deep vein thrombosis, nomogram, predictive model

## Abstract

**Objective:**

To identify independent risk factors for isolated distal deep vein thrombosis (IDDVT) following endovascular treatment (EVT) in patients with acute ischemic stroke (AIS), and to establish a practical and accurate nomogram-based prediction model.

**Methods:**

A retrospective review was performed on 263 AIS patients who underwent EVT at the First Affiliated Hospital of Ningbo University between September 2022 and September 2024. Patients were divided into IDDVT and non-IDDVT groups based on postoperative ultrasound findings. Baseline characteristics were compared, and univariate and multivariate logistic regression analyses were conducted to determine independent predictors of IDDVT. A nomogram was constructed based on regression results. Model performance was evaluated using the area under the receiver operating characteristic curve (AUC), calibration curves, and decision curve analysis (DCA).

**Results:**

Among 263 patients, 45 (17.1%) developed IDDVT. Multivariate logistic regression revealed that elevated D-dimer levels, decreased lower limb muscle strength, prior stroke history, and intracranial hemorrhagic transformation after EVT were independent risk factors (all *p* < 0.05). The nomogram incorporating these predictors demonstrated excellent discrimination, with an AUC of 0.903 (95% CI: 0.856–0.950), sensitivity of 86.7%, and specificity of 87.2%. Calibration and DCA confirmed good accuracy and clinical applicability.

**Conclusion:**

Elevated D-dimer levels, reduced lower limb strength, history of stroke, and postoperative hemorrhagic transformation serve as critical warning indicators for IDDVT after EVT in AIS patients. The nomogram developed in this study provides a high-precision tool for individualized risk prediction, supporting early risk stratification and preventive decision-making in clinical practice.

## 1 Introduction

Acute ischemic stroke (AIS) is characterized by high incidence, disability, recurrence, and mortality rates, posing a serious threat to human health and quality of life (1). For AIS caused by large vessel occlusion (LVO), endovascular treatment (EVT) has become the standard of care to improve functional outcomes (2). However, the management of complications following EVT remains a major challenge for optimizing patient recovery.

Deep vein thrombosis (DVT) of the lower extremities is one of the most common complications after stroke, with an incidence ranging from 11.4 to 49.6% (3). Isolated distal deep vein thrombosis (IDDVT) refers to thrombosis confined to the intramuscular venous plexus or tibial and peroneal veins below the popliteal vein, accounting for 20–50% of all DVT cases (4). Although often asymptomatic, IDDVT carries a risk of proximal propagation and subsequent pulmonary embolism (PE), one of the leading causes of death in stroke patients (5, 6). The first DVT event typically occurs within 2–7 days after stroke onset, coinciding with the perioperative phase of EVT (7). Post-stroke hemiplegia and immobilization further increase the risk of DVT in this population (8).

Several general venous thromboembolism (VTE) risk assessment tools, such as the Caprini and Padua scores, are currently used in clinical practice. However, these models were primarily designed for medical or surgical inpatients and fail to adequately account for stroke-specific factors (9), such as neurological deficits and stroke severity, limiting their predictive accuracy in AIS patients. Although some nomogram models have been developed to predict DVT after stroke in general populations (10, 11), few studies have specifically targeted patients undergoing EVT, a subgroup characterized by both high risk and clinical complexity. These patients not only experience stroke-related complications but also face additional risks associated with endovascular procedures and postoperative management.

Therefore, this study aimed to retrospectively analyze risk factors for IDDVT in AIS patients after EVT and to establish a precise, visualized nomogram prediction model. This model is expected to facilitate early identification of high-risk patients and to support individualized preventive strategies in clinical practice.

## 2 Materials and methods

### 2.1 Study population

Clinical data of AIS patients admitted to the Department of Neurology, First Affiliated Hospital of Ningbo University, between September 2022 and September 2024 were retrospectively collected. All patients received EVT, and the study protocol was approved by the institutional ethics committee. Inclusion criteria: (1) acute onset, with AIS caused by intracranial large- or medium-vessel occlusion confirmed by CT/MRI and CTA/MRA; (2) EVT performed within 24 h of onset; (3) complete clinical and imaging data. Exclusion criteria: (1) pre-existing DVT before EVT; (2) severe cardiac, hepatic, renal, or coagulation dysfunction; (3) active malignancy; (4) long-term use of oral anticoagulants; (5) hospitalization <7 days or incomplete data (patients with hospital stays shorter than 7 days were excluded to ensure a minimum of 1 week of in-hospital observation for DVT detection and complete data collection). IDDVT was defined as thrombosis confined to the calf deep veins distal to the popliteal vein (e.g., anterior tibial, posterior tibial, peroneal, or muscular veins), without involvement of the popliteal or more proximal veins. Based on postoperative Doppler ultrasonography within 7 days, 263 patients were enrolled and divided into IDDVT (*n* = 45) and non-IDDVT (*n* = 218) groups. IDDVT is defined as thrombosis limited to the deep veins of the calf distal to the popliteal vein (such as the anterior tibial, posterior tibial, and peroneal veins), without involvement of the popliteal vein or more proximal deep veins (12). Management strategies for IDDVT included therapeutic anticoagulation as the first-line treatment when not contraindicated, physical therapy measures (such as limb elevation and restricted activity) for patients with bleeding risk, and consideration of inferior vena cava (IVC) filter placement in high-risk cases where anticoagulation was not feasible.

### 2.2 Data collection

Clinical data were obtained from electronic medical records, including: (1) Baseline characteristics: age, sex, and comorbidities (hypertension, diabetes, hyperlipidemia, coronary artery disease, atrial fibrillation, and prior stroke). (2) Clinical and laboratory parameters: occlusion site (ICA, MCA, ACA, BA, VA), lower limb muscle strength [graded 0–5 using the standard Medical Research Council (MRC) scale, assessed by board-certified neurologists in the Department of Neurology], postoperative hemorrhagic transformation, and infections (pulmonary, urinary). Laboratory tests—including blood count, renal/hepatic function, lipid profile, coagulation function, D-dimer, and C-reactive protein (CRP)—were collected within 48 h after EVT to reflect the early postoperative status. (3) Outcome assessment: IDDVT occurrence confirmed by bilateral Doppler ultrasound within 7 days after EVT.

### 2.3 Statistical analysis

All analyses were performed using R (version 4.2.1). Continuous variables were presented as mean ± SD or median (IQR) and compared using Student’s *t*-test or Mann–Whitney U test. Categorical variables were presented as n (%) and compared using chi-square or Fisher’s exact test. Variables with *p* < 0.1 in univariate analysis were entered into multivariate logistic regression using forward stepwise selection. Odds ratios (ORs) with 95% confidence intervals (CIs) were calculated. A nomogram was constructed based on independent predictors. Model performance was assessed using ROC analysis (AUC), calibration plots, and DCA. Statistical significance was defined as *p* < 0.05.

## 3 Results

### 3.1 Patient characteristics

A total of 263 AIS patients treated with EVT were included, with a mean age of 67.3 ± 13.1 years; 170 (64.6%) were male. IDDVT occurred in 45 patients (17.1%). Compared with the non-IDDVT group, patients with IDDVT had higher rates of coronary artery disease, prior stroke, atrial fibrillation, pulmonary and urinary infections, and intracranial hemorrhagic transformation. They also had significantly higher D-dimer and CRP levels, and poorer lower limb strength (more patients with grade 0–2 paralysis). Details are summarized in Table 1.

**Table 1:** Demographic and clinical characteristics of patients with and without IDDVT after endovascular treatment for AIS.

Characteristics	Total (*n* = 263)	No IDDVT (*n* = 218)	IDDVT (*n* = 45)	*P*-value
Demographic characteristics				
Age, years	67.30 ± 13.12	66.61 ± 13.35	70.77 ± 11.41	0.073
Gender				0.512
Male	170 (64.6%)	139 (63.8%)	31 (68.9%)	
Female	93 (35.4%)	79 (36.2%)	14 (31.1%)	
Clinical parameters				
Hypertension	153 (58.2%)	123 (56.4%)	30 (66.7%)	0.205
Diabetes mellitus	57 (21.7%)	45 (20.6%)	12 (26.7%)	0.372
Coronary heart disease	21 (8.0%)	13 (6.0%)	8 (17.8%)	0.014
History of stroke	6 (2.3%)	1 (0.5%)	5 (11.1%)	<0.001
Smoking	72 (27.4%)	58 (26.6%)	14 (31.1%)	0.537
Pulmonary infection	4 (1.5%)	1 (0.5%)	3 (6.7%)	0.002
Urinary infection	7 (2.7%)	3 (1.4%)	4 (8.9%)	0.018
Hyperlipemia	15 (5.7%)	12 (5.5%)	3 (6.7%)	0.760
Atrial fibrillation	71 (27.0%)	53 (24.3%)	18 (40.0%)	0.031
Hypertensive heart disease^‡^	27 (38.0%)	20 (37.7%)	7 (38.9%)	
Ischemic heart disease^‡^	15 (21.1%)	11 (20.8%)	4 (22.2%)	
Valvular heart disease^‡^	7 (9.9%)	5 (9.4%)	2 (11.1%)	
Cardiomyopathy^‡^	3 (4.2%)	1 (1.9%)	2 (11.1%)	
Undetermined etiology^‡^	16 (22.5%)	13 (24.5%)	3 (16.7%)	
Laboratory results				
TG (mmol/L)	1.4 (0.9, 2.1)	1.3 (0.9, 2)	1.58 (0.9, 2.8)	<0.303
TC (mmol/L)	3.8 ± 1.1	3.84 ± 1.04	3.75 ± 1.13	0.649
HDL (mmol/L)	1.02 (0.87, 1.23)	1.01 (0.82, 1.23)	1.03 (0.9, 1.23)	0.369
LDL (mmol/L)	2.46 ± 0.81	2.46 ± 0.79	2.46 ± 0.9	0.754
Hcy (umol/L)	13.95 ± 8.51	13.82 ± 8.51	14.58 ± 8.55	0.498
Fast glucose (mmol/L)	6.86 (5.68, 8.47)	6.65 (5.68, 8.5)	7.12 (5.4, 7.99)	0.808
UA, (umol/L)	266.52 ± 121.8	262.5 ± 120.02	285.98 ± 129.74	0.196
D-dimer (ng/mL)	910 (461.5, 2475.5)	784.5 (417.5, 1738.5)	3,680 (1,060, 6,536)	<0.001
SCr (umol/L)	81.6 ± 70.8	81.65 ± 76.59	81.4 ± 30.74	0.377
CRP (mg/L)	18.67 (7.69, 36.70)	15.84 (7.05, 28.79)	38.08 (17.00, 63.89)	<0.001
TP (g/L)	60.3 ± 6.5	60.1 ± 6.1	61.4 ± 8.4	0.357
Alb (g/L)	35.1 ± 4.5	35.1 ± 4.2	34.7 ± 5.4	0.257
Occlusion location				0.167
ICA	93 (35.4%)	76 (34.9%)	17 (37.8%)	
MCA	116 (44.1%)	100 (45.9%)	16 (35.6%)	
ACA	1 (0.3%)	1 (0.5%)	0 (0%)	
BA	50 (19.0%)	40 (18.3%)	10 (22.2%)	
VA	3 (1.1%)	1 (0.5%)	2 (4.4%)	
Intracerebral hemorrhage	51 (19.4%)	29 (13.3%)	22 (48.9%)	<0.001
Lower limb muscle strength				<0.001
level 0	11 (4.2%)	4 (1.8%)	7 (15.6%)	
level 1	35 (13.3%)	22 (10.1%)	13 (28.9%)	
level 2	61 (23.2%)	54 (24.8%)	7 (15.6%)	
level 3	77 (29.3%)	68 (31.2%)	9 (20.0%)	
level 4	58 (22.1%)	50 (22.9%)	8 (17.8%)	
level 5	21 (8%)	20 (9.2%)	1 (2.2%)	
Mortality	56 (21.3%)	39 (18.9%)	17 (37.8%)	0.006
Neurological causes of death^†^	43 (76.8%)	30 (76.9%)	13 (76.5%)	
Non-neurological causes of death^†^	13 (23.2%)	9 (23.1%)	4 (23.5%)	

IDDVT, isolated distal deep venous thrombosis; ^‡^Percentages are calculated among patients with atrial fibrillation; ^†^Percentages are calculated among deceased patients.

### 3.2 Independent risk factors for IDDVT

Variables with *p* < 0.1 in the univariate analysis were included in the multivariate logistic regression model. The results showed that D-dimer levels (OR = 1.0005, 95% CI: 1.0003–1.0007, *p* < 0.001), lower limb muscle strength (OR = 0.564, 95% CI: 0.379–0.815, *p* = 0.003), history of stroke (OR = 114.5, 95% CI: 2.23–5.888, *p* = 0.008), and intracranial hemorrhagic transformation (OR = 0.125, 95% CI: 0.044–0.329, *p* < 0.001) were independent risk factors for the development of IDDVT (Table 2).

**Table 2:** Predictive factors for IDDVT among AIS patients after endovascular treatment.

Intercept and variable	β	Odds ratio (95%CI)	*P*-value
Lower limb muscle strength	−0.572	0.564 (0.379–0.815)	0.003
D-dimer	0.0005	1.0005 (1.0003–1.0007)	<0.001
Intracerebral hemorrhage	−2.0819	0.125 (0.044–0329)	<0.001
History of stroke	4.74	114.5 (2.23–5,888)	0.008

β is the regression coefficient; CI, confidence interval.

### 3.3 Construction and performance evaluation of the nomo predictive model

**Figure 1:**
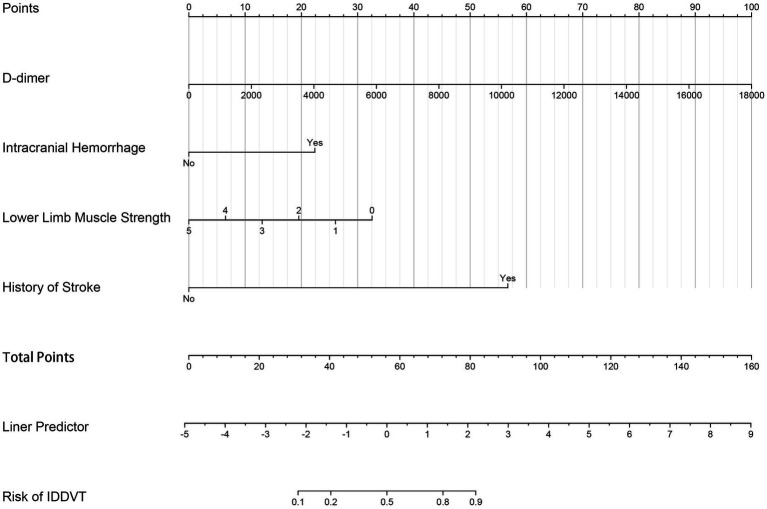
Nomogram for predicting the risk of IDDVT after endovascular therapy in AIS patients. For each patient, sum the points assigned for all six predictors on the top point scale. The total points are then projected to the bottom axis to determine the corresponding probability of IDDVT risk.

**Figure 2:**
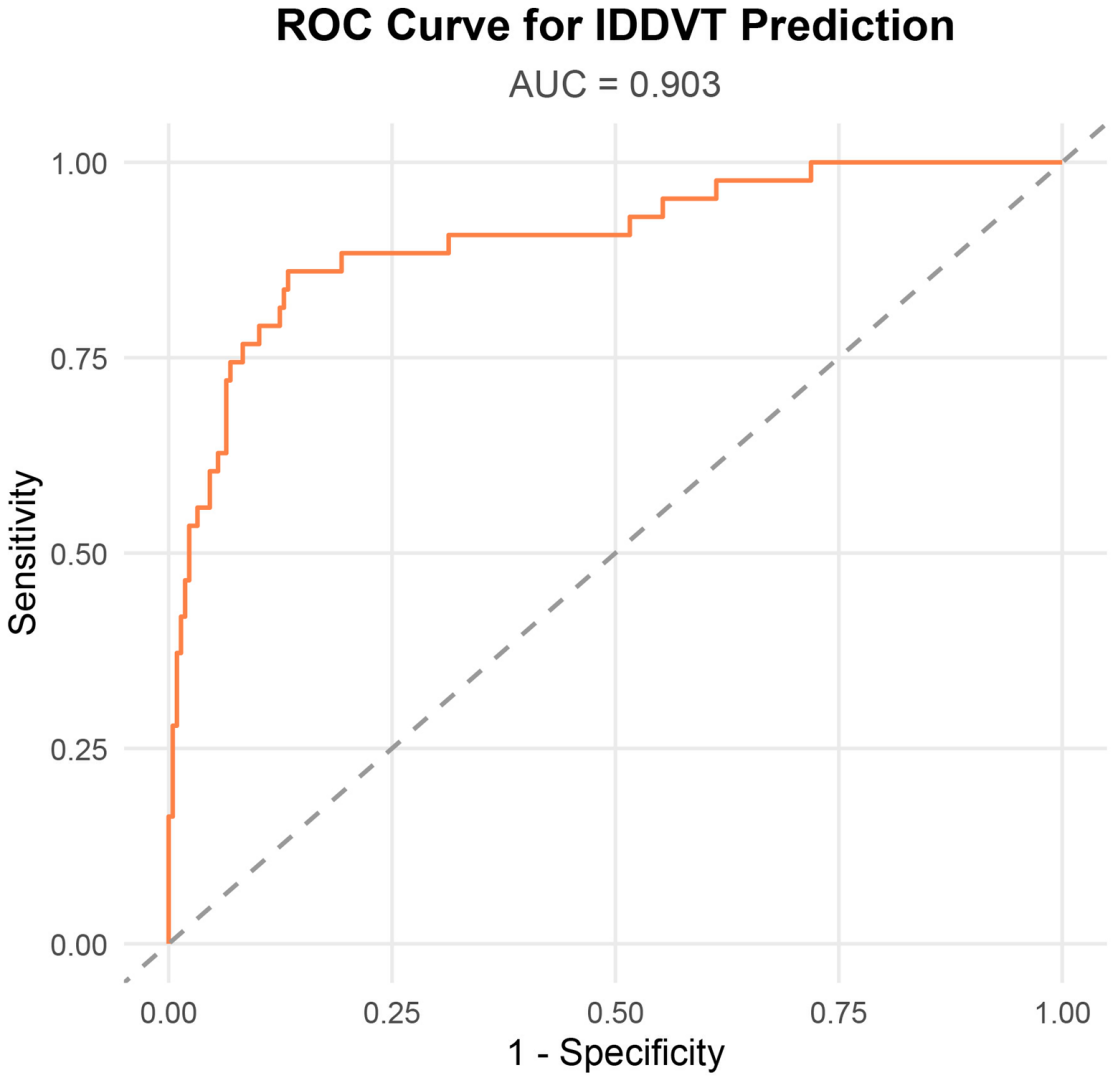
Receiver operating characteristic curves for IDDVT model. IDDVT, isolated distal deep venous thrombosis; ROC, receiver operating characteristic.

**Figure 3:**
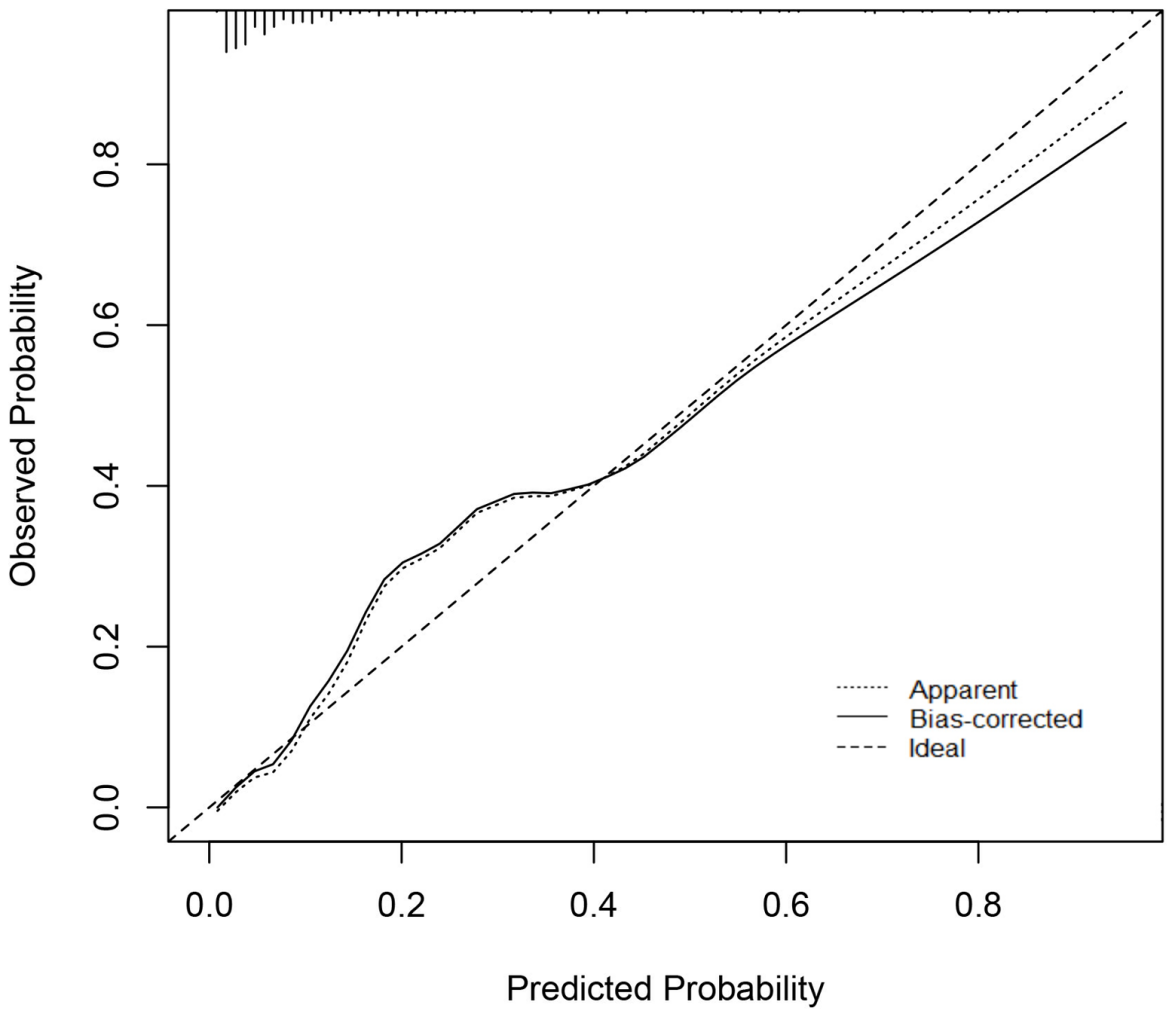
Calibration curve of the IDDVT nomogram prediction in this study. The x-axis represents the predicted risk of IDDVT, while the y-axis indicates the actual diagnosed incidence. The diagonal dashed line illustrates the ideal prediction of a perfect model. The solid line represents the performance of the nomogram; closer alignment with the diagonal dashed line indicates better predictive accuracy.

**Figure 4:**
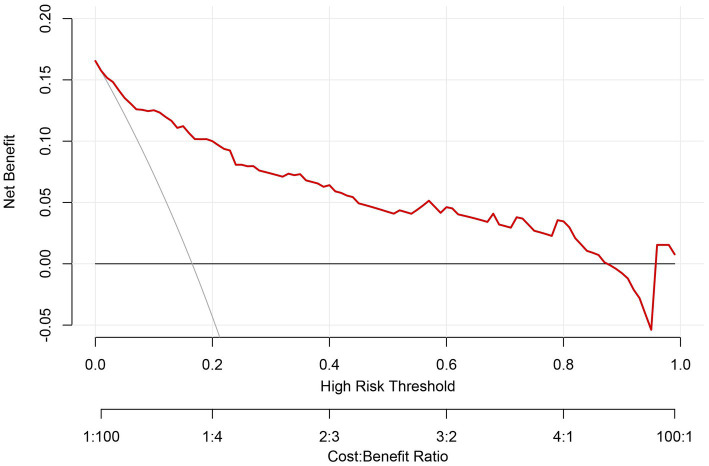
Decision curve analysis for the IDDVT nomogram. The red line represents the IDDVT risk model, the fine line represents the predict-all-patients as IDDVT, and the thick line represents the predict-none-patients as IDDVT.

**Figure 5:**
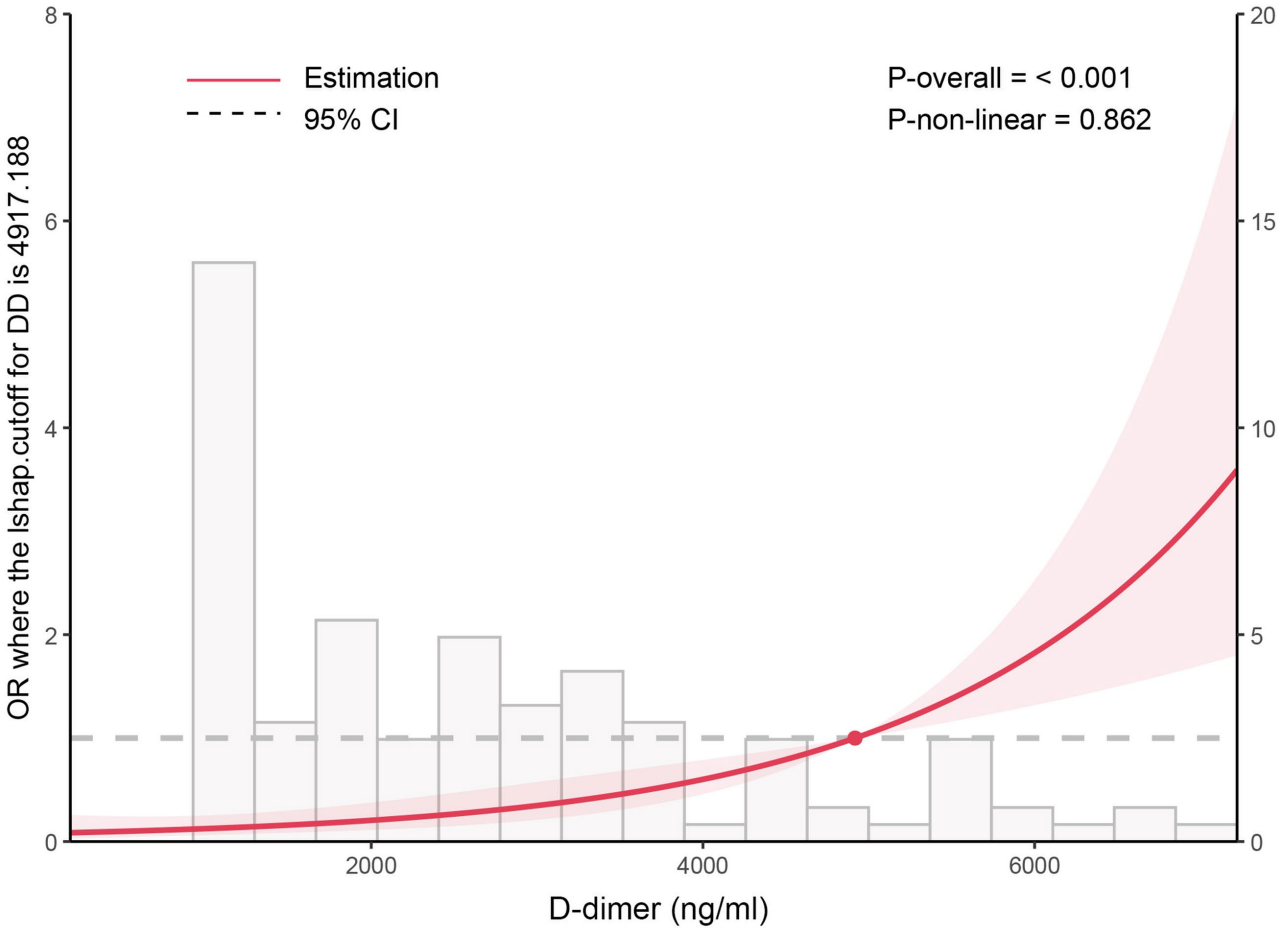
Restricted cubic spline curve illustrating the relationship between D-dimer levels and the occurrence of IDDVT after endovascular therapy in AIS patients.

Based on the four independent risk factors identified above, a nomogram model was developed to predict the risk of IDDVT (Figure 1). To evaluate the predictive performance of the model, a receiver operating characteristic (ROC) curve was plotted (Figure 2). The results showed an area under the curve (AUC) of 0.903 (95% CI: 0.856–0.950), indicating excellent discriminative ability. At the optimal cutoff point (where the Youden index is maximized), the model achieved a sensitivity of 86.7% and a specificity of 87.2%. At this cutoff, the model yielded a PPV of 58.2%, an NPV of 96.9%, and an overall diagnostic accuracy of 87.1% (TP = 39, TN = 190, FP = 28, FN = 6) (Supplementary Table S1). The Hosmer–Lemeshow test yielded a *p*-value of 0.672, suggesting no significant deviation between predicted and observed probabilities, which confirms good calibration of the model (Figure 3). Decision curve analysis (Figure 4) demonstrated that, across a wide threshold probability range of 2 to 80%, the use of this nomogram provided higher net benefit than both the “treat-all” and “treat-none” strategies, supporting its clinical utility. Furthermore, a restricted cubic spline plot indicated a gradually increasing risk of IDDVT with higher D-dimer levels (Figure 5).

## 4 Discussion

EVT has markedly improved outcomes in AIS, yet management of postoperative complications remains crucial for long-term recovery. This study is the first to systematically investigate IDDVT in AIS patients after EVT, reporting an incidence of 17.1% and establishing a high-accuracy prediction model (AUC = 0.903).

This study identified four independent risk factors significantly associated with IDDVT through multivariate analysis, the underlying mechanisms of which warrant further investigation. First, D-dimer level was the biomarker with the strongest predictive value. As a specific degradation product of cross-linked fibrin, elevated D-dimer directly reflects the simultaneous activation of both the coagulation and fibrinolytic systems (13, 14). Stroke, as an acute stress event, can trigger a systemic inflammatory response, activate endothelial cells and platelets, and induce a hypercoagulable state (15). Our study found that D-dimer levels were significantly higher in the IDDVT group than in the non-IDDVT group, which is consistent with previous findings in general stroke populations (8, 16) and various thrombotic diseases (17). In patients undergoing endovascular treatment, procedural manipulation may cause minor endothelial injury, further exacerbating the hypercoagulable state. Therefore, dynamic monitoring of D-dimer should not only be used as an auxiliary diagnostic tool but also serve as a critical early warning signal for initiating or escalating preventive measures.

Lower limb muscle weakness represents a hemodynamic mechanism, as hemiplegia impairs the calf-muscle pump (18), leading to venous stasis—one of Virchow’s triad. Severe paralysis was strongly associated with IDDVT (19), underscoring the importance of early physical prophylaxis and rehabilitation when feasible.

A history of prior stroke was another strong predictor, likely reflecting underlying systemic atherosclerosis, atrial fibrillation, and cumulative functional impairment (20). These patients may require intensified monitoring and preventive strategies.

Finally, postoperative hemorrhagic transformation was revealed as a novel and clinically significant risk factor. To prevent hematoma expansion, clinicians often delay or withhold antithrombotic therapy (21), inadvertently creating a window of increased thrombotic risk. Moreover, hemorrhagic transformation worsens neurological deficits and prolongs immobilization, compounding venous stasis. This finding highlights the need for balancing hemorrhage management with non-pharmacological VTE prevention.

Previous studies have further emphasized the prognostic significance of intraventricular extension of intracerebral hemorrhage. In particular, Arboix et al. (22) demonstrated that intraventricular hemorrhage is a strong and independent determinant of poor outcome in patients with intracerebral hemorrhage, likely reflecting increased intracranial pressure, impaired cerebrospinal fluid circulation, and secondary inflammatory injury. Intraventricular extension is therefore considered a marker of hemorrhage severity and is closely associated with neurological deterioration and mortality. In the present study, hemorrhagic transformation after EVT was recorded as a binary variable, and intraventricular extension was not analyzed separately due to the retrospective design and limited sample size. Nevertheless, patients with hemorrhagic transformation—particularly those with more extensive bleeding—tended to have more severe neurological deficits and prolonged immobilization, which may indirectly contribute to an increased risk of IDDVT. Future studies with larger cohorts and detailed hemorrhage subtyping are warranted to further clarify the impact of intraventricular extension on thrombotic complications after EVT.

In addition, we observed that patients in the IDDVT group were more likely to present with multiple comorbidities such as hypertension, atrial fibrillation, and a history of prior stroke, compared to those without IDDVT. This suggests that the IDDVT cohort represented a clinically more fragile population with greater baseline vascular burden and systemic disease complexity. This observation aligns with findings from Cheng et al. (16), who reported that acute ischemic stroke patients with early IDDVT had significantly higher rates of atrial fibrillation, stroke-associated pneumonia, and malignancy, suggesting a strong association between comorbidity load, systemic inflammation, and in-hospital thrombosis risk. Atrial fibrillation–related ischemic stroke is typically cardioembolic in nature. Patients with atrial fibrillation in our cohort showed a poorer short-term prognosis. Consistent with this observation, previous studies have demonstrated that cardioembolic stroke is associated with worse early outcomes compared with other ischemic stroke subtypes, including higher early mortality (23).

Another pivotal contribution of this study lies in the development of a nomogram model incorporating four key variables. This model not only demonstrated excellent discriminative ability (AUC = 0.903), significantly outperforming many general-purpose (24) or other models designed for general stroke populations (18, 25, 26), but also offers an intuitive and user-friendly format suitable for rapid bedside assessment. By plotting the corresponding values of the four indicators on the nomogram, clinicians can readily obtain a quantitative, individualized probability of IDDVT risk. This risk-stratification management approach carries substantial clinical implications. For instance, patients identified as high-risk by the model could be assigned to an intensified prevention protocol, which may include increased frequency of lower extremity vascular ultrasound screenings, prioritized application of intermittent pneumatic compression devices, and earlier initiation of pharmacological prophylaxis in the absence of contraindications. Conversely, for low-risk patients, unnecessary preventive measures and their associated potential risks (such as bleeding) can be avoided, thereby facilitating optimized allocation of medical resources and advancing personalized precision medicine.

Overall, future studies focusing on cardioembolic stroke, especially atrial fibrillation–related stroke, may provide deeper insights into IDDVT risk after EVT. Prospective multicenter research incorporating detailed cardiac evaluation and longer-term outcomes is warranted.

## 5 Limitation

Limitations include the retrospective, single-center design, which may restrict generalizability. Although the sample size was adequate for model development, external validation in multi-center prospective cohorts is warranted. Furthermore, perioperative management details and genetic predispositions were not assessed. The study focused on short-term IDDVT events, without evaluating long-term outcomes or recurrence. Extended follow-up studies are needed.

## Conclusion

Elevated D-dimer, reduced lower limb strength, prior stroke, and hemorrhagic transformation after EVT are independent predictors of IDDVT in AIS patients. A nomogram based on these factors demonstrated excellent predictive accuracy and clinical utility, supporting early identification of high-risk patients and guiding individualized preventive strategies.

## Data Availability

The original contributions presented in the study are included in the article/Supplementary material, further inquiries can be directed to the corresponding authors.
