# Supplementary material for: Risk factors and nomogram prediction model for isolated distal deep vein thrombosis after endovascular treatment in acute ischemic stroke patients
Source: Front Neurol. 2026 Feb 24;17:1701326. doi: 10.3389/fneur.2026.1701326 (PMC12974093; doi:10.3389/fneur.2026.1701326)
Supplement: Supplementary file 1 [file Table_1.docx]

**Supplementary Table S1. Diagnostic performance of the nomogram-based multivariable model for predicting IDDVT**

| **Diagnostic metric** | **Value** |
| --- | --- |
| Area under the ROC curve (AUC) | 0.903 |
| Optimal cutoff (Youden index) | 0.23 |
| Sensitivity, % | 86.7 |
| Specificity, % | 87.2 |
| Positive predictive value (PPV), % | 58.2 |
| Negative predictive value (NPV), % | 96.9 |
| Diagnostic accuracy, % | 87.1 |

**Footnote：**
The optimal cutoff value was determined by maximizing the Youden index. Diagnostic performance metrics were calculated based on the multivariable nomogram model.
